# Incidence and Predictors of Cardiovascular Complications and Death after Vascular Surgery

**DOI:** 10.5935/abc.20150113

**Published:** 2015-11

**Authors:** Luciana Andrea Avena Smeili, Paulo Andrade Lotufo

**Affiliations:** 1 Hospital Universitário da USP, São Paulo, SP – Brazil; 2 Hospital das Clínicas da FMUSP, São Paulo, SP – Brazil

**Keywords:** Cardiovascular Diseases/complications, Vascular Diseases/surgery, Mortality, Postoperative Complications, Risk Assessment, Postoperative/mortality

## Abstract

**Background:**

Patients undergoing arterial vascular surgery are considered at increased risk for post-operative complications.

**Objective:**

To assess the incidence and predictors of complications and death, as well as the performance of two models of risk stratification, in vascular surgery.

**Methods:**

This study determined the incidence of cardiovascular complications and deaths within 30 days from surgery in adults. Univariate comparison and logistic regression assessed the risk factors associated with the outcomes, and the receiver operating characteristic (ROC) curve assessed the discriminatory capacity of the revised cardiac risk index (RCRI) and vascular study group of New England cardiac risk index (VSG-CRI).

**Results:**

141 patients (mean age, 66 years; 65% men) underwent the following surgeries: carotid (15); lower limbs (65); abdominal aorta (56); and others (5). Cardiovascular complications and death occurred within 30 days in 28 (19.9%) and 20 (14.2%) patients, respectively. The risk predictors were: age, obesity, stroke, poor functional capacity, altered scintigraphy, surgery of the aorta, and troponin change. The scores RCRI and VSG-CRI had area under the curve of 0.635 and 0.639 for early cardiovascular complications, and 0.562 and 0.610 for death in 30 days.

**Conclusion:**

In this small and selected group of patients undergoing arterial vascular surgery, the incidence of adverse events was elevated. The risk assessment indices RCRI and VSG-CRI did not perform well for complications within 30 days.

## Introduction

Every year, 250 million major surgeries are performed worldwide, with mortality of 1% and morbidity of 5%. Patients surviving post-operative complications usually have functional limitations and reduced survival^1-4^.

Patients undergoing vascular surgery are considered at increased risk for post-operative cardiovascular adverse events because: many risk factors that contribute to vascular disease also contribute to coronary artery disease (CAD), such as diabetes and smoking; CAD symptoms can be blurred by low functional capacity; and vascular surgeries can be associated with significant blood volume fluctuations and thrombogenicity^5-7^.

How to perform a more accurate risk assessment of those patients remains uncertain^8-12^.

Two studies (Table I) have validated the most often used risk assessment models in our practice: revised cardiac risk index (RCRI), derived from a heterogeneous population of patients, of which only a small percentage underwent vascular surgery; and the vascular study group of New England cardiac risk index (VSG-CRI), specific for vascular surgery. However, none of them has approached mortality in their outcomes^12,13^.

**Table 1:** Revised Cardiac Risk Index (RCRI)^13^ and Vascular Study Group of New England Cardiac Risk Index (VSG-CRI)^12^

Variable RCRI	Points	
CAD	1
HF	1
Stroke or transient ischemic accident	1
Insulin-dependent diabetes	1
Creatinine ≥ 2.0	1
High-risk surgery: abdominal, thoracic or suprainguinal vascular surgery	1
**Variable VSG-CRI**	**Points**
Age > 80 years	4
Age from 70 to 79 years	3
Age from 60 to 69 years	2
CAD	2
HF	2
COPD	2
Creatinine > 1.8	2
Smoking	1
Insulin-dependent diabetes	1
Long-term beta-blocker use	1
History of angioplasty or coronary revascularization	-1 (protective)

Points/risk – RCRI: 0 = low; 1-2 = moderate; >2 = high; VSG-CRI: 0-4 = low; 5-6 = moderate; > 6 = high risk. CAD: Coronary artery disease; HF: Heart failure; COPD: Chronic obstructive pulmonary disease.

This study was aimed at assessing the epidemiological characteristics and clinical outcome of a cohort of patients undergoing vascular surgery, as well as identifying possible predictors of adverse events.

## Methods

This study prospectively assessed a sample of patients admitted to the vascular surgery ward of the Hospital das Clínicas de São Paulo from August 2008 to January 2010.

The inclusion criteria were: age ≥ 18 years; patients of both sexes; and elective hospital admission for open vascular surgery or endovascular surgery of occlusive atherosclerotic disease or degenerative aneurysmal disease.

The exclusion criteria were: need for emergency surgery; varicose vein stripping; thromboembolectomy; vascular access formation; and refusal to provide written informed consent.

This study was approved by the Ethics Committee.

The primary objectives were:

a) to assess the incidence of cardiovascular complications, all-cause death and combined outcome (cardiovascular complications and/or death) within 30 days from surgery;

b) to identify possible predictors for those adverse events;

c) to analyze whether RCRI and VSG-CRI have good accuracy to estimate the occurrence of cardiovascular complications and total death.

Cardiovascular complications were defined as non-fatal myocardial infarction, decompensated heart failure, significant arrhythmia and stroke.

The secondary objectives were:

a) to assess the causes of death, classifying them into cardiovascular and non-cardiovascular;

b) to assess the incidence of non-cardiovascular complications: wound infection; septic shock; hemorrhagic shock; severe renal failure; respiratory complications; venous thromboembolism; amputation; and reoperation.

The patients were classified according to their surgeries as follows: carotid surgery; lower limbs surgery; abdominal aorta surgery; and other surgeries.

The following variables were recorded: related to the patient (history, physical exam, laboratory tests, electrocardiography and imaging); related to the surgical procedure; and occurrence of adverse outcomes within 30 days from surgery.

### Statistical analysis

To assess risk factors, bivariate comparisons of the variables selected were performed in patients with and without the outcomes. Logistic regression was performed, and the odds ratio (OR) and 95% confidence interval for the risk of the outcome in question were calculated.

The discriminatory capacity of the risk assessment models (RCRI and VSG-CRI) was evaluated by use of the receiver operating characteristic (ROC) curve. The statistical software SPSS, version 17, was used.

## Results

### Pre-operative clinical variables

This study assessed 141 patients, whose mean age was 66 years, 62 patients were 70 years or older, and 92 patients were of the male sex. Table II shows the prevalence of previous diseases.

**Table 2:** Clinical characteristics of the sample

Age	< 70 years	≥ 70 years
	79 (56.0%)	62 (44.0%)
Sex	female (%)	male (%)
	49 (34.8%)	92 (65.2%)
Antecedents	absent	present
Coronary insufficiency	85 (60.3%)	56 (39.7%)
HF	64 (45.4%)	77 (54.6%)
Dyslipidemia	64 (45.4%)	77 (54.6%)
Arterial hypertension	23 (16.3%)	118 (83.7%)
Stroke	110 (78.0%)	31 (22.0%)
Peripheral arterial insufficiency with claudication	28 (19.9%)	113 (80.1%)
Peripheral arterial insufficiency with trophic changes	98 (69.5%)	43 (30.5%)
COPD	107 (75.9%)	34 (24.1%)
Diabetes	87 (61.7%)	54 (38.3%)
Renal failure	73 (51.8%)	68 (48.2%)
Smoking habit	30 (21.3%)	111 (78.7%)
Revascularization or angioplasty	119 (84.4%)	22 (15.6%)
Low weight	134 (95%)	7 (5%)
Overweight	91 (64.6%)	50 (35.5%)
Obesity	126 (89.3%)	15 (10.6%)
FC	≥ 4 MET	< 4 MET
	59 (41.8%)	82 (58.2%)
	No	Yes
Statin use	16 (11.3%)	125 (88.7%)
Beta-blocker use	44 (31.2%)	97 (68.8%)
Acetylsalicylic acid use	14 (9.9%)	127 (90.1%)

HF: Heart failure; COPD: Chronic obstructive pulmonary disease; FC: Functional capacity.

Almost half of the individuals had renal failure, defined as creatinine clearance lower than 60 mL/min, and 19 patients (13%) had creatinine clearance lower than or equal to 40 mL/min. The NT pro-BNP was lower than 100 ng/L in 30%, and greater than 500 ng/L in 30% of the patients.

A significant change in the electrocardiogram was observed in 59 (42%) patients, and in the chest radiography, suggestive of heart failure, in 57 (40%) patients.

Doppler echocardiography was performed in 68% of the patients, 86% of whom had an ejection fraction greater than 50%, while 7% had it lower than 40%.

Of the 73% undergoing scintigraphy, almost 40% had a defect, persistent low uptake being observed in 33, transient low uptake in 13, and extensive transient low uptake in 2.

Regarding perioperative risk assessment, 132 patients (93.6%) were considered ASA II, and 9 (6.4%), ASA III.

The RCRI classified 6 patients (4.3%) as at low risk, 63 (44.7%) as at moderate risk, and 72 (51%) as at high risk. According to the VSG-CRI, 34 (24.1%) patients were classified as at low risk, 44 (31.2%) as at moderate risk, and 63 (44.7%) as at high risk.

### Variables related to surgery

The 141 patients underwent the following surgeries: carotid surgery, 15 (106%); lower limbs, 65 (46.1%); abdominal aorta, 56 (39.7%); and other surgeries, 5 (3.5%).

Open and endovascular surgeries were performed in 58% and 42% of the patients, respectively. The anesthesia was general or combined in 91.5%. The mean times of anesthesia and orotracheal intubation were 330 min and 875 min, respectively. Blood derivatives were required by 61 patients (43.3%), while vasoactive drugs, by 57 patients (40.4%). Troponin was collected in 128 patients (90.8%), 31 of whom (22%) had it altered.

### Events in the post-operative period up to 30 days

Cardiovascular complications were observed in 28 patients (19.9%), 20 of whom (14.2%) died within that period. Combined complications occurred in 39 patients (27.7%).

Early cardiovascular complications were as follows: myocardial infarction, 18 patients; decompensated heart failure, 12; stroke, 3; and arrhythmia, 6.

Of the 20 patients dying early, 5 had a cardiovascular cause among the major causes of death (myocardial infarction and/or cardiogenic shock). A non-cardiovascular cause was identified among the major causes of death in 18 patients, being the major cause of death in 15 patients. Septic shock was diagnosed in 10 patients, hemorrhagic shock, in 10, and pulmonary complications, in 4. Thus, only 25% of the deaths had a cardiovascular cause, the non-cardiovascular being the major cause of death in 75%.

Non-cardiovascular complications were observed in 55 (39%) patients as follows: pulmonary, 15; renal failure, 15; septic shock, 16; wound infection, 19; venous thromboembolism, 3; and reoperations, 27.

### Predictors of events

The variables with p < 0.05 were as follows:

-**for early cardiovascular complication:** age (p = 0.028), stroke (0.045), obesity (0.025), scintigraphy with transient alteration (0.021), open surgery (p = 0.024), time of anesthesia (p = 0.08), and use of blood derivatives (p = 0.045);
-**for death:** poor functional capacity (p = 0.026), troponina change (p = 0.002), and times of anesthesia (p = 0.010) and of intubation (p = 0.019). Symptomatic peripheral arterial insufficiency correlated inversely with that outcome (p = 0.026).
-**for early combined complication:** age (p = 0.044), times of anesthesia (p = 0.000) and of intubation (p = 0.018), use of blood derivatives (p = 0.005) and of vasoactive drugs (p = 0.029), and aorta surgery (p = 0.01). Surgery of the lower limbs (p = 0.003) correlated inversely with that outcome.

Assessment of the accuracy of the indices RCRI and VSG‑CRI in the sample

**Figure 1:**
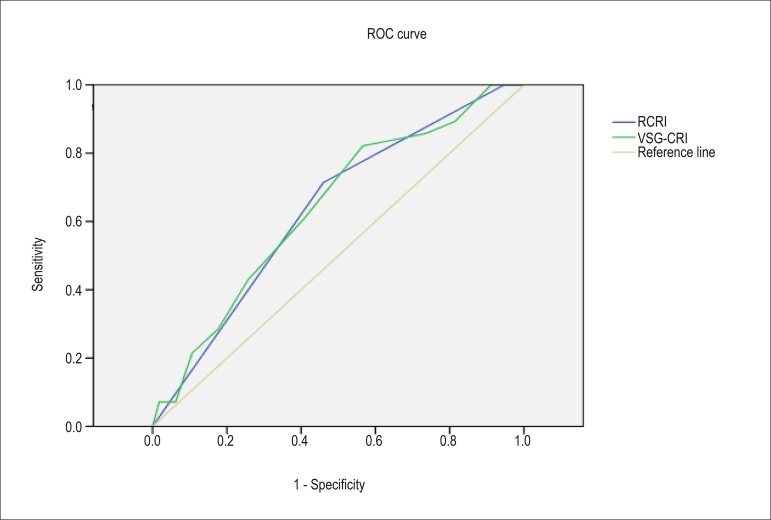
Receiver operating characteristic (ROC) curve for the outcome cardiovascular complications. RCRI: Revised cardiac risk Index; VSG-CRI: Vascular study group of New England cardiac risk index.

The AUC of RCRI and VSG-CRI were, respectively: for early cardiovascular complications, 0.635 and 0.639 (Figure 1); for total mortality, 0.562 and 0.610; and for early combined complication, 0.618 and 0.622.

## Discussion

### Clinical characteristics of the sample

Table III compares the clinical characteristics found in the studies by Parmar et al^14^, Bertges et al^12^, Eagle et al^5^ and Meltzer et al^15^ for vascular surgery, and in the study by Lee et al^13^ for general surgery^5,12,15^.

**Table 3:** Major predictors of perioperative morbidity and mortality

Predictor/ Reference	ASA PS 1941 ^38^	Goldman ^8^	Detsky ^9^	Eagle ^5^	Lee ^13^	Bartel, Reilly, Older ^31,32^^,33^	Karthikeyan ^39^	Bertges ^12^	Parmar ^14^	Bradbury ^34^	Gupta ^18^	Meltzer ^15^
GSH	+										+	
Age		+	+	+				+	+	+	+	+
CAD		+	+	+	+			+	+			+
HF		+	+	+	+			+				
AoS		+	+									
Arrhythmias		+	+									
Hypoxemia		+	+									
Hypercapnia		+	+									
Hypokalemia		+	+									
Acidosis		+	+									
Renal failure		+	+		+			+		+	+	+
Liver disease		+	+									
Bedridden patient		+	+									
ITPS		+									+	
ITTS		+	+		+						+	
Aorta surg.		+	+	+	+						+	+
EMS		+	+									
Scintigraphy				+								
Diabetes				+	+			+		+		
VS				+	+						+	
Stroke					+							
FC						+					+	+
COPD								+				
Smoking								+		++		
BB								+				
Revasc ^k^								-				
SPAI^l^										+		+
BMI^n^										+		
BNP							+					

GSH: Patient's general state of health; CAD: Coronary artery disease; HF: Heart failure; AoS: Aortic stenosis; ITPS: Intraperitoneal surgery; ITTS: Intrathoracic surgery; EMS: Emergency surgery; VS: Vascular surgery; FC: Functional capacity; COPD: Chronic obstructive pulmonary disease; BB: Beta-blocker; Revasc.: Myocardial revascularization; SPAI: Symptomatic peripheral arterial insufficiency; BMI: Body mass index; BNP: Brain natriuretic peptide. + Risk factor; – Protective factor.

The following variables were similar in the studies by Parmar et al^14^, Bertges et al^12^ and Eagle et al^5^: age, sex and comorbidities (stroke, chronic obstructive pulmonary disease, diabetes and renal failure)^5,12,14^.

Comparing with the mean of the four studies on vascular surgery, our study showed a higher prevalence of symptomatic peripheral arterial insufficiency, heart failure and arterial hypertension; lower prevalence of previous myocardial revascularization; and high use of acetylsalicylic acid, statin and beta-blocker.

More than half of our sample was classified as having poor functional capacity (58%), similarly to the population of the study by Eagle et al^5^, but higher than that reported by Meltzer et al^15^.

Most of our patients were considered at high or moderate risk according to the RCRI and VSG-CRI scores.

We had more endovascular surgeries, a factor that could reduce our risk of events^16,17^. However, choosing endovascular intervention over open surgery could have been based on a high estimated surgical risk.

Another factor that increased the complexity of our sample was the smaller number of carotid surgeries, considered of intermediate risk^10^, and predominance of more complex procedures involving the aorta and lower limbs.

Our prevalence of ischemia in myocardial scintigraphy was lower than that reported by Eagle et al^5^.

Due to the nature of our patients, elevated prevalence of chronic diseases and clinical evidence of severe ischemia of the lower limbs, in addition to poor functional capacity, we cannot generalize our findings to the general population submitted to vascular surgery.

### Cardiovascular events and/or death in up to 30 days

A high rate of early events was observed, higher than that reported in the literature.

### Cardiovascular events in up to 30 days

Within 30 days from surgery, the 20% cardiovascular complication rate was much greater than the 2.5% reported by Lee et al^13^ for general surgery and the 6.3% reported by Bertges et al^12^ for vascular surgery. That rate was equivalent to the one reported for abdominal aorta open surgery (19.3- 22.6%) by Bertges et al^12^.

The 12% incidence of infarction exceeded the 4.5% reported by Eagle et al^5^, whose sample had a high prevalence of ischemia on scintigraphy, but that study has not used troponin as a diagnostic criterion of infarction. That might have underestimated the number of events found.

### Total death within 30 days from surgery

In the post-operative period of non-cardiac surgery, the prevalence of total death is low in non-selected populations and in general surgery, ranging from 0.02% to 2.3%. That number, however, is higher when the patient’s or surgery’s complexity increases, reaching 4% for colectomy, 2.9% for revascularization of lower limbs, and as much as 70% for the general surgery of an ASA V patient^8,15,18,19^.

Regarding total mortality, our 14% exceeded the expected. Previous studies have estimated mortality of 1.5% for carotid surgery, 4.1%-7% for surgery of the lower limbs, and 3.9%-9% for aorta surgery^7,20,21^.

In a large study (6839 patients) with population characteristics similar to ours, mortality within 30 days after amputation of the lower limbs ranged from 9% to 12%^22^.

The total mortality found in this study can reflect the severely-ill sample and its high cardiac complication rate. Mortality after post-operative events is high: Gupta et al^18^ have reported that 61% of the patients developing infarction and cardiac arrest died within 30 days.

Similarly, that general mortality can also reflect the high incidence of non-cardiovascular complications (27.7%) in up to 30 days. Meltzer et al^15^ have reported a high mortality rate for patients with complications: 19% for infectious complications, 33% for cardiac complications, and 42% for pulmonary complications.

### The outcome ‘cardiac death’

The cardiac death rate was 3.5%, greater that estimated for general surgery in non-selected patients (0.54%-1.8%). Goldman et al^8^ have reported that the cardiac death rate can be very elevated, depending on the patient’s clinical characteristics, reaching 56% for those classified as class IV^8,18^.

### Outcome ‘cardiac death’ and/or cardiac complication

In our study, 33 patients (23.4%) had that outcome. Studies assessing cardiac death and/or cardiac complication in patients with risk factors for ischemic cardiac disease or with known heart disease and general surgery have reported values ranging from 7.9% to 18%^9,23^.

### Non-cardiovascular morbidity in up to 30 days

The non-cardiac complication rate found in this study, almost 30%, was higher than the 22% reported by Gawande et al. for colectomy, a surgery known for its high incidence of complications, and the 19% reported by Meltzer et al. for the revascularization surgery of the lower limbs^15,19^.

In 2005, Khuri et al^24^ carried out a multicenter prospective study with almost 106,000 patients and reported that post-operative adverse events were more important than pre- and intra-operative variables to determine survival after general surgery. The presence of a complication within the first 30 post-operative days, regardless of the patient’s pre-operative risk, reduced survival by 69%. The presence of pulmonary complication in that study reduced long-term survival by 87%, and the presence of wound complication reduced survival by 42%^24^.

That important finding shows the long-term repercussion of post-operative morbidity. In the presence of cardiovascular complications, one may assume that they will interfere with long-term survival. However, for non-cardiovascular complications, such as wound infection and pulmonary complications, the presence of an exacerbated persistent inflammatory state is suggested to reduce survival^24^.

Thus, strategies are required to estimate the risk for non-cardiovascular complications, as well as to monitor and prevent them, because their importance should not be underestimated, despite the smaller number of studies and predictive indices so far available regarding that outcome.

### Predictors of events within 30 days

Regarding the variables related to patients, the following predictors are compatible with literature data: age; stroke; poor functional capacity; and transient low uptake on scintigraphy^5,8,12,13,15,18,25-28^.

The variable obesity requires larger prospective studies to confirm its relationship with that outcome. Differently from our findings, Bradburry et al. have identified lower 2-year survival in low-weight patients with peripheral arterial disease and indication for surgery^28^.

The variable ‘symptomatic peripheral arterial insufficiency’ showed a negative correlation with death within 30 days, differently from that expected^15,28^ and independently of the type of surgery (open or endovascular).

We observed a tendency towards fewer combined events in patients undergoing surgery of the lower limbs and more combined events in patients undergoing abdominal aorta surgery. Several previous studies have shown the relationship between surgery of the aorta and the risk for post-operative adverse events^8,9,13,18^. The lower rate of events found in the group of lower limb surgery might reflect the inclusion of surgeries of heterogeneous complexities (amputation, revascularization, popliteal aneurysm).

The following surgery-related factors that correlated with cardiac complications and death reflected the magnitude of surgical stress and tissue injury, independently of underlying diseases and surgery type, although interdependently: time of anesthesia, need for blood derivatives and vasoactive drugs, and time of intubation.

Several indices considered surgery magnitude in the risk assessment of post-operative death, such as Possum (1998), the Surgical Risk Scale (SRS - 2002) and the ISIS score (Identification of Risk in Surgical Patients - 2010)^28,29,30^.

The time of anesthesia might have been a predictor and marker of events, reflecting surgery complexity and possible intra-operative events that prolong that time.

Studies have shown the importance of intra-operative hemodynamic stability. The Apgar score used three parameters: heart rate, arterial blood pressure and amount of blood loss^19^. The variables ‘vasoactive drugs’ and ‘blood derivatives’ might have been markers of those hemodynamic changes.

As already shown, troponin correlated with death within 30 days. Devereaux et al^31^ have shown a mortality rate increase within 30 days from 1.9%, for negative troponin in the post-operative period, to 9.3%, for troponin greater than or equal to 0.30 ng/mL.

### Accuracy of the risk scores RCRI and VSG-CRI

The accuracy of RCRI to discriminate between patients at high or low risk for perioperative cardiac events is considered moderate (AUC of 0.74) for general surgery. However, its performance has been considered low in the subgroup of patients undergoing vascular surgery in the original study (AUC of 0.54 for open surgery of abdominal aorta)^13,32^.

In a systematic review performed by Ford et al^32^, the performance of RCRI for general surgery was similar to that of the original study (AUC of 0.75); however, for vascular surgery, that index did not show good discriminatory capacity (AUC of 0.64).

Similarly, our study showed a good discriminatory capacity of RCRI to predict cardiovascular events in up to 30 days (AUC of 0.635).

One limitation of the study by Lee et al^13^, and also of our study, was to assess the predictive performance of an index only by use of AUC^32^.

VSG-CRI is considered to perform better than RCRI as a predictor of cardiovascular events after vascular surgery (AUC of 0.71). However, it did not show good performance in our study (AUC of 0.639)^12^.

The use of any predictive index requires it to maintain its discriminative capacity in different populations, geographical sites and time. Further studies are necessary to conclude on the value of VSG-CRI among us.

Neither RCRI nor VSG-CRI were good predictors of early death and early combined complications (cardiovascular and/or death) in our study.

More studies are required to test new indices including variables such as age, body mass index, peripheral arterial insufficiency magnitude, functional capacity and myocardial scintigraphy.

In addition to a more accurate pre-operative index for clinical risk assessment, another score could be used right after surgery, as has been suggested by Gawande et al. in their proposal to build an Apgar score^19^.

The Apgar score considers higher heart rate and lower arterial blood pressure, in addition to the amount of intra-operative blood loss, showing good correlation with cardiac and non-cardiac complications and death within 30 days^19^.

Based on this study’s findings, we suggest the development of an immediate post-operative score including the following data: times of anesthesia and of intubation, and need for blood derivatives and vasoactive drugs.

Because the factors related to patients are not the only responsible for adverse events, a two-time perioperative risk assessment, pre-operative and at the end of surgery, might enhance the accuracy to identify patients in need for greater attention, monitoring and post-operative tests.

## Conclusions

Our sample had a high rate of cardiovascular events and total death in up to 30 days.

We observed a greater number of non-cardiovascular deaths than that of cardiovascular deaths, and many non-cardiovascular complications.

Age, stroke, obesity, ischemic alterations on myocardial scintigraphy, aorta surgeries, low functional capacity and troponin changes were identified as risk factors for events in up to 30 days. The risk assessment indices RCRI and VSG-CRI proved not to be good predictors of events.
